# Effects of functional electrical stimulation on cognition rate and gait in neurological patients during single- and dual-task walking

**DOI:** 10.1038/s41598-025-98755-w

**Published:** 2025-04-19

**Authors:** Niklas Bleichner, Daniel W. W. Heitzmann, Jennifer Raynaud, Andreas Stähle, Claudia Weichold, Merkur Alimusaj, Cornelia Putz, Frauke Nees, Herta Flor, Sebastian I. Wolf

**Affiliations:** 1 Clinic for Orthopedics, Heidelberg University Hospital, 69118 Heidelberg, Germany; 2 Institute of Neuropsychology and Clinical Psychology, Central Institute of Mental Health Mannheim- University of Heidelberg, 68159 Mannheim, Germany; 3 Institute of Medical Psychology and Medical Sociology, University Medical Center Schleswig-Holstein- Kiel University, 24105 Kiel, Germany

**Keywords:** Neurological disorders, Foot drop, Dual-task, Functional electrical stimulation, Cognition, Gait, Neurological disorders, Rehabilitation

## Abstract

People with neurological disorders and foot drop may suffer from cognitive-motor interference during walking. Functional Electrical Stimulation (FES) targets foot drop during gait but its effects on cognition remain underexplored. Fifteen individuals (4 males, 11 females; mean age 35.5 ± 12.5 years) with various neurological disorders, who had been using FES for at least three months, were recruited from our outpatient clinic. Subjects were assessed during walking with and without FES (FES CONDITION) and under single- and dual-task walking (TASK). The dual-task consisted of counting backwards from a number near 100 in steps of seven while walking. Cognition rate served as outcome parameter, with high values indicating subjects could efficiently maintain both cognitive task performance and walking speed. A linear mixed model analysis was conducted with FES CONDITION and TASK as fixed and SUBJECTS as random effects. The cognition rate during dual-task walking was significantly better with FES vs. without. FES showed minor effects on dorsiflexion in swing but larger effects on overall gait, as reflected in walking speed, step length and step width. While dual-task led to inferior results in gait, FES counteracted this effect and improved cognition rate. These findings suggest that FES not only addresses gait pattern and stability but also frees cognitive resources for walking. This shift in focus may enhance environmental awareness, social interaction or multitasking and thereby improving overall independence and quality of life, which is particularly relevant for patients with neurological disorders and an increased risk of falls.

## Introduction

Persons with neurological disorders often show altered walking patterns characterized by higher gait variability^1^ and an increased incidence of falls compared to age-matched healthy individuals, with step length being a primary determinant^2^.Conditions such as cerebral palsy, spinal cord injuries, traumatic brain injuries, stroke, multiple sclerosis, and hereditary spastic paraplegia disrupt upper motor neuron pathways, thus leading to altered motor control^3^.

A common impairment among these conditions is a lesion of dorsiflexor muscles and a resulting foot drop, which decreases toe clearance during swing phase and thereby increases risk of toe dragging and falling^4^.Ankle-foot orthoses (AFOs) are widely used to prevent foot drop by stabilizing the foot. However, they typically limit push-off power^5^, restrict ankle movement to some degree and show drawbacks regarding size, weight, appearance and usable footwear^6^.

Since its introduction in 1961, functional electrical stimulation (FES) has become more and more popular as an alternative treatment for foot drop. With intact lower motor neurons and peripheral nerves, impaired muscles can be reactivated^7^.In the literature the effects of FES are differentiated into immediate effects of FES (orthotic) and therapeutic effects, which are described as long-term carry-over effects of FES that are present even when the device is not worn^6,8,9^.

Despite a direct (bio-)mechanical effect of AFOs, FES has the advantage of re-engaging the patient’s own muscle function, thus promoting neuroplasticity when used over a longer period^9^. This neuroplasticity is assumed to be the leading mechanism to achieve therapeutic effects^10^ with patients showing greater neural plasticity experience better therapeutic effects^11^.Additionally, FES could potentially help reintegrate the affected limb into the body schema, enabling patients to regain or enhance body ownership and spatial awareness^12,13^. Nonetheless structural but also dynamic anatomical deformities might lead to a necessity of additional conventional orthosis such as foot orthosis or an additional stabilisation of the ankle by a stabilising AFO. Combinations of both FES and conventional orthosis might address these issues.

However, the effects of FES on gait function vary in the literature. While some authors have shown that dorsiflexion during swing phase is improved with FES^14–17^ the evidence remains limited^18^. FES has been associated with improvements in walking speed similar to those provided by AFOs^9,19–23^. Although no improvement has been seen in individuals with cerebral palsy (orthotic effect)^17^ FES has also been linked to greater subjective experience regarding stability during gait, quality of gait pattern, effort of walking^21^ and satisfaction^23^ compared to AFOs. In addition a reduction in gastrocnemius spasticity^16^, an increase in M. tibialis anterior volume and strength^24^, and a decrease in toe dragging and falls^25^ has been shown.

The main goal of FES in foot drop management is to achieve an adequate foot lift during swing phase. While studies demonstrate significant improvements, a recent pilot study of our group, showed faster walking speed over time but revealed no significant improvement in dorsiflexion during swing^26,27^. Nevertheless, both the literature and our pilot data indicate high patient satisfaction, with reports of enhanced comfort, stability, and safety, making FES a preferred choice among patients^21^.

An underexplored but potentially important aspect of FES therapy is its impact on real-life multitasking demands. Cognitive-motor interference occurs frequently in everyday situations, which often require the simultaneous performance of cognitive and motor tasks to effectively interact with the environment. Given that attention is a limited resource, splitting it between two tasks can lead to decreased performance in one or both tasks^28,29^. This is especially relevant for patients with neurological disorders, where higher gait variability^1^, an increased risk of falling^30^, and dual-task interferences can significantly impact walking speed and cognitive performance, with weaker cognitive functioning often leading to slower walking speeds^31^.

Thus, for individuals with neurological conditions and foot drop, a rehabilitation tool addressing both gait and cognitive performance may be superior for everyday interactions. This study aims to evaluate the long-term effects of FES on gait kinematics, spatio-temporal parameters, and cognitive performance during single-task (ST) and dual-task (DT) walking, hypothesizing that FES will enhance both gait and cognitive performance.

## Methods

### Subjects

Subjects were recruited in the Orthotics and Prosthetics Department of the Orthopedic Hospital of Heidelberg University and provided written informed consent to participate in this study. The study received approval from the Ethics Committee of the Medical Faculty of Heidelberg University (S-503/2022) and all methods were carried out in accordance with relevant guidelines and regulations (Declaration of Helsinki).

First inclusion criterion was to have used one of the FES systems Evomove (Evomotion GmbH, Lueneburg, Germany) or L300Go (Otto Bock HealthCare Deutschland GmbH, Duderstadt, Germany) as continuously for at least the past three months. Both devices use accelerometer and gyroscope data to detect gait phases and are individually adjusted by a certified local orthotist to ensure optimal outcomes for each subject.

Further inclusion criteria were: ability to provide consent for study participation, presence of a neurological disorder with upper motor neuron lesions leading to foot drop, age between 18 and 70 years and the ability to walk unaided for at least 15 min. Exclusion criteria were an equinus foot deformity exceeding 5° of plantarflexion, any previous surgical interventions, or botulinum toxin injections in the leg muscles within the past six months. Table 1 summarises information on all subjects, including demographic information and diagnosis.

## Study design

**Fig. 1:**
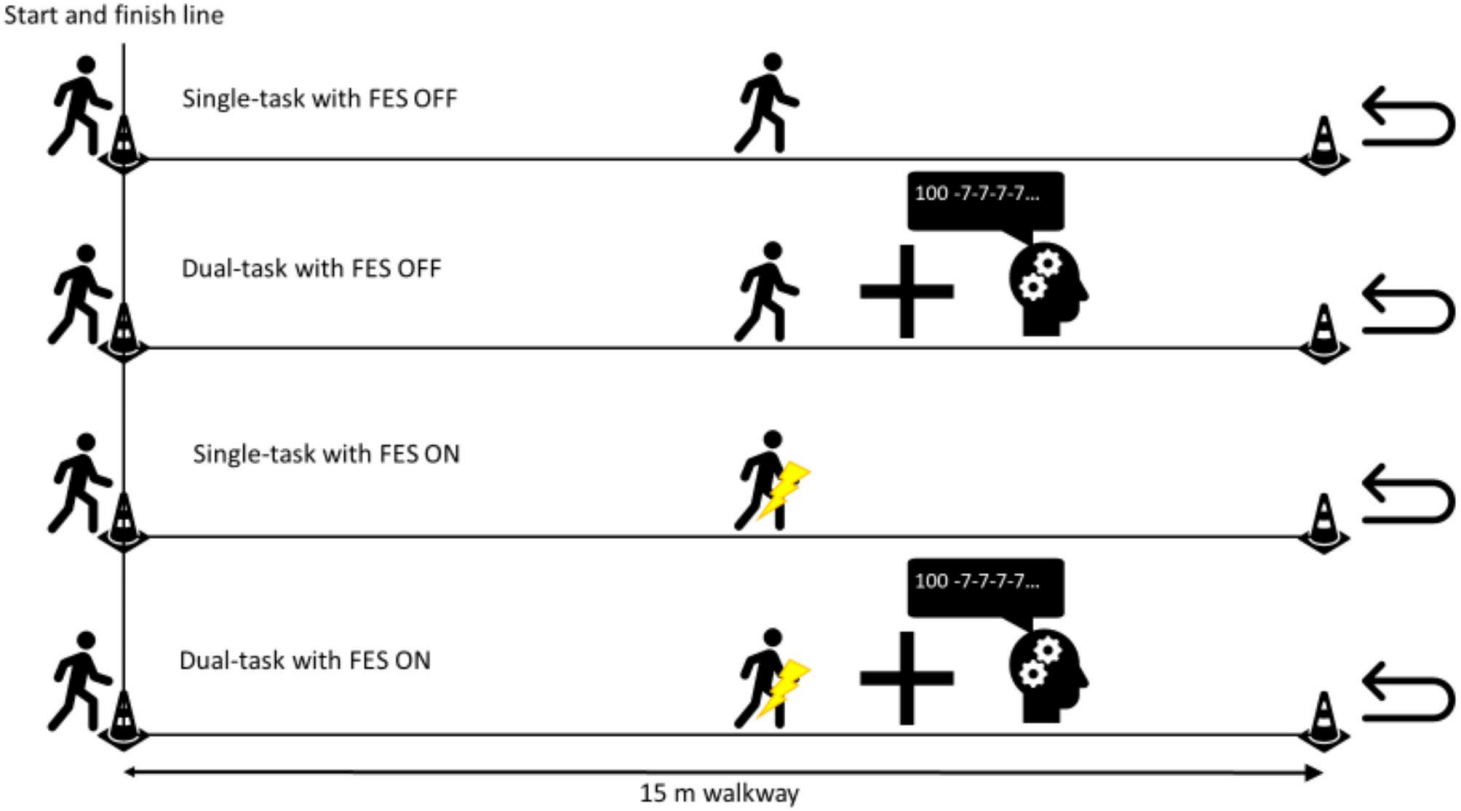
The four walking conditions: without FES during single- and dual-task walking, followed by with FES during single- and dual-task walking. The cognitive task required counting backward from near 100 in steps of seven. (FES: functional electrical stimulation).

This within-subject observational study employed conventional 3D gait analysis and clinical examination to assess lower extremity joint range of motion, spasticity, strength, and selective motor control. Gait kinematics and spatio-temporal parameters were evaluated when the participants were walking on level ground with standardized footwear (Deichmann SE, Essen, Germany). The main study consisted of a more extensive measurement protocol including the following conditions first without FES, followed by with FES: standard level ground, single-task and dual-task walking followed by walking up and down a 10° inclined ramp. The four walking conditions—without FES vs. with FES and single-task (ST) vs. dual-task (DT) walking—were conducted in a fixed sequence: without FES during single- and dual-task walking, followed by with FES during single- and dual-task walking (Fig. 1). Subjects walked at a self-selected speed towards a cone at the end of the 15-meter walkway and returned to the starting position (total distance = 30 m). Subjects could freely choose whether to turn right or left around the cone.

For the dual-task condition, subjects completed one practice trial followed by two recorded trials. The additional cognitive task required counting backward from a randomised starting number near 100 in steps of seven. It has been shown that the task is difficult enough to alter gait and that the method is reliable for repeated trials^32,33^. In the familiarisation trial, the starting number was set to 100, while for the two subsequent actually recorded trials, starting numbers were randomised near 100 to minimise learning effects and only numbers were chosen which avoid multiples of seven to prevent calculation advantages. If an error occurred, subjects were instructed to continue counting, with the next correct step of seven being counted as correct. The correct and total numbers of responses were extracted from the recorded video.

For 3D-motion capture, a Vicon System (Oxford, UK) with consisting of twelve cameras (T40s, 120 Hz) was utilised. Reflective, skin mounted markers were placed on each subject according to the Plug-In-Gait (PiG) protocol^34–36^ with an additional marker on the hallux to calculate toe clearance. Ground reaction forces were measured by two force plates (1080 Hz, AMTI, Inc., Watertown, MA, USA) embedded inconspicuously in the walkway. Joint kinematics and kinetics were calculated using the conventional inverse dynamics approach with PiG software (Vicon, Oxford, UK) and normalized to body weight, resulting in joint moments expressed in Nm/kg and powers in W/kg.

## Outcome measures

### Gait parameters

Hip, knee, and ankle kinematics were calculated and the focus was set onto those variables influencing toe clearance including ankle dorsiflexion, and knee flexion of the affected leg during swing phase, and hip abduction of the contralateral leg during stance. General gait function was evaluated with spatio-temporal parameters, including walking speed, step length, and step width.

### Cognition rate

The cognition rate was calculated by dividing the number of correct answers by the total number of answers, then dividing by the walking time^37^. With high cognition rate indicating that subjects can efficiently maintain both cognitive task performance and walking speed, while a low cognition rate suggests either less correct answers relative to total answers, slower walking speed, or both.

1 $$\:Cognition\:rate=\frac{\frac{correct\:answers}{total\:answers}}{time}\times\:100\%$$

### Statistical analyses

Linear mixed model analysis was conducted to evaluate the effects of FES CONDITION (without FES vs. with FES), TASK (ST vs. DT), and their interaction (FES CONDITION *TASK) on gait parameters. In the mixed model, FES CONDITION and TASK were treated as fixed effects, while SUBJECTS were considered as a random effect to account for inter-individual variability. P-values, β-coefficients, and 95% confidence intervals (CIs) were calculated for each dependent variable to determine the main effects of FES CONDITION and TASK as well as their interaction. For better clarity, the results tables for the linear mixed model (LMM) are divided into two: one table for means (SDs) and p-values, and another for β-coefficients and 95% CIs.

Additionally, isolated comparisons of the effects of FES CONDITION and TASK were performed using Bonferroni-corrected paired t-tests. For spatio-temporal parameters, isolated comparisons for FES CONDITION included ST: without FES vs. with FES, and DT: without FES vs. with FES. For TASK, comparisons included without FES: ST vs. DT and with FES: ST vs. DT. The cognition rate during DT walking was also compared between without FES and with FES.

The significance level was set at *p* < 0.05, and statistical analyses were performed using SPSS (version 27; SPSS, Chicago, IL, USA). Descriptive statistics included mean values with standard deviations and mean differences.

## Results

### Demographics

As part of a larger study, fifteen subjects with neurological impairments at first motor neuron level—such as cerebral palsy, incomplete spinal cord injury, multiple sclerosis, traumatic brain injury, and hereditary spastic paraplegia—who exhibited foot drop and had used functional electrical stimulation for at least three months were included in this analysis. Table 1 presents their demographical data.

**Table 1:** Demographics of included patients (SD: standard deviation).

ID	Diagnosis	Gender	Age [years]	Height [cm]	Weight [kg]
1	Cerebral Palsy	m	18	183	66
2	Cerebral Palsy	f	25	173	72
3	Cerebral Palsy	m	26	172	80
4	Cerebral Palsy	m	26	172	63
5	Cerebral Palsy	f	28	153	49
6	Cerebral Palsy	f	33	154	49
7	Hereditary spastic paraplegia	m	63	179	71
8	Incomplete spinal cord injury	f	29	170	83
9	Incomplete spinal cord injury	f	33	171	64
10	Incomplete spinal cord injury	f	38	168	92
11	Incomplete spinal cord injury	f	39	174	74
12	Incomplete spinal cord injury	f	48	194	104
13	Incomplete spinal cord injury	f	53	158	110
14	Multiple Sclerosis	f	49	162	66
15	Traumatic brain injury	f	26	168	51
	Mean (SD)	4 m, 11 f	35.5 (± 12.5)	170.1 (± 10.8)	73 (± 18.5)

### Spatio-temporal parameters

The linear mixed model revealed significant main effects for both FES CONDITION (β = 0.06, *p* = 0.012, 95% CI = 0.01 to 0.11) and TASK (β = -0.09, *p* = 0.001, 95% CI = 0.04 to 0.14) on walking speed, but no significant interaction between them (Tables 2 and 3).

**Table 2:** Mean values (SD) of gait variables with linear mixed model results, with TASK and FES CONDITION as fixed effects and SUBJECTS as random effects (all variables except of max. Hip abduction stance are from the affected leg).

*N* = 15	Without FES				With FES				*p*-value		
Kinematics	ST		DT		ST		DT		TASK	FES CONDITION	TASK* FES CONDITION
Max. dorsiflexion stance [°]	17.8	(± 8.0)	17.7	(± 8.3)	18.7	(± 9.3)	18.3	(± 9.5)	0.543	0.146	0.734
Dorsiflexion at heel-strike [°]	-2.2	(± 8.6)	-1.2	(± 8.3)	-0.7	(± 8.8)	0.2	(± 8.8)	0.292	0.089	0.940
Max. dorsiflexion swing [°]	1.5	(± 8.2)	2.2	(± 8.8)	2.6	(± 9.5)	3.5	(± 9.4)	0.256	0.150	0.840
Max. knee flexion swing [°]	55.4	(± 9.3)	54.3	(± 9.7)	56.5	(± 9.8)	54.9	(± 10.6)	**0.024**	0.136	0.654
Max. hip abduction stance [°]	5.8	(± 5.5)	5.7	(± 5.7)	6.3	(± 6.5)	5.9	(± 6.2)	0.347	0.195	0.650
Min. toe clearance mid-swing [mm]	36.8	(± 6.1)	38.0	(± 5.8)	37.9	(± 7.7)	38.4	(± 7.5)	0.640	0.472	0.781
Max. toe clearance swing [mm]	127.4	(± 36.4)	123.7	(± 37.4)	141.1	(± 39.5)	133.7	(± 39.8)	0.125	**0.002**	0.678
Spatio-temporal parameters											
Speed [m/s]	1.17	(± 0.25)	1.08	(± 0.23)	1.24	(± 0.20)	1.14	(± 0.22)	**0.001**	**0.012**	0.911
Step length [m]	0.66	(± 0.09)	0.63	(± 0.08)	0.69	(± 0.08)	0.66	(± 0.09)	**0.003**	**0.002**	0.891
Step width [cm]	9.29	(± 3.80)	9.89	(± 4.01)	8.38	(± 3.90)	8.77	(± 3.72)	0.308	**0.019**	0.685

Significant values are in bold.

SD: standard deviation, FES: functional electrical stimulation, ST: single-task, DT: dual-task.

**Table 3:** Linear mixed model results with TASK and FES CONDITION as fixed effects and SUBJECTS as random effects (all variables except of max. Hip abduction stance are from the affected leg).

*N* = 15	TASK			FES CONDITION			TASK* FES CONDITION		
Kinematics	β	95% CI		β	95% CI		β	95% CI	
Max. dorsiflexion stance [°]	-0.39	1.67	-0.89	0.94	2.23	-0.34	0.31	1.51	-2.12
Dorsiflexion at Heel-Strike [°]	0.91	0.81	-2.64	1.49	3.21	-0.24	0.09	2.35	-2.53
Max. dorsiflexion swing [°]	0.87	0.66	-2.40	1.11	2.64	-0.42	-0.22	2.38	-1.95
Max. knee flexion swing [°]	**-1.61**	**3.00**	**0.22**	1.05	2.43	-0.34	0.44	1.52	-2.40
Max. hip abduction stance [°]	-0.36	1.13	-0.41	0.50	1.27	-0.27	0.25	0.84	-1.34
Min. toe clearance mid-swing [mm]	0.66	2.16	-3.47	1.01	3.83	-1.80	0.55	3.39	-4.48
Max. toe clearance swing [mm]	-5.85	13.39	-1.69	**12.11**	**19.65**	**4.57**	2.18	8.36	-12.73
Spatio-temporal parameters									
Speed [m/s]	**-0.09**	**0.14**	**0.04**	**0.06**	**0.11**	**0.01**	0.00	0.07	-0.06
Step length [m]	**-0.03**	**0.04**	**0.01**	**0.03**	**0.05**	**0.01**	0.00	0.02	-0.03
Step width [cm]	0.39	0.37	-1.14	**-0.91**	**-0.15**	**-1.66**	0.22	0.85	-1.28

Significant values are in bold.

FES: functional electrical stimulation; β: regression coefficient, CI: confidence interval

With FES, walking speed increased by 0.06 m/s during single-task walking and by 0.07 m/s during dual-task walking. Dual-task walking led to a reduction in walking speed by 0.09 m/s, regardless of without or with FES (Table 4).

**Table 4:** Mean values (SD) of spatio-temporal parameters with bonferroni corrected paired t-test results (*N* = 15; affected leg for step length).

Without FES: ST vs. DT	ST without FES		DT without FES		Mean diff.	*p*-value	95 CI	
Speed [m/s]	1.17	(± 0.25)	1.08	(± 0.23)	-0.09	**0.001**	-0.05	-0.14
Step length [m]	0.66	(± 0.09)	0.63	(± 0.08)	-0.03	**0.005**	-0.01	-0.04
Step width [cm]	9.29	(± 3.80)	9.89	(± 4.01)	0.60	0.108	1.35	-0.15

With FES: ST vs. DT	ST with FES		DT with FES					
Speed [m/s]	1.24	(± 0.20)	1.14	(± 0.22)	-0.09	**0.001**	-0.06	-0.12
Step length [m]	0.69	(± 0.08)	0.66	(± 0.09)	-0.03	**0.007**	-0.01	-0.05
Step width [cm]	8.38	(± 3.90)	8.77	(± 3.72)	0.39	0.218	1.03	-0.26

ST: without FES vs. with FES	ST without FES		ST with FES					
Speed [m/s]	1.17	(± 0.25)	1.24	(± 0.20)	0.06	**0.035**	0.12	0.01
Step length [m]	0.66	(± 0.09)	0.69	(± 0.08)	0.03	**0.006**	0.05	0.01
Step width [cm]	9.29	(± 3.80)	8.38	(± 3.90)	-0.91	**0.034**	-0.08	-1.74

DT: without FES vs. with FES	DT without FES		DT with FES					
Speed [m/s]	1.08	(± 0.23)	1.14	(± 0.22)	0.07	**0.007**	0.11	0.02
Step length [m]	0.63	(± 0.08)	0.66	(± 0.09)	0.03	**0.001**	0.04	0.01
Step width [cm]	9.89	(± 4.01)	8.77	(± 3.72)	-1.12	**0.007**	-0.37	-1.88
Cognitive performance								
Cognition rate	3.47	(± 0.82)	3.72	(± 0.82)	0.25	**0.005**	0.40	0.09

Significant values are in bold.

SD: standard deviation, FES: functional electrical stimulation, ST: single-task, DT: dual-task, diff: difference, CI: confidence interval

The effects on step length followed a similar pattern. Both FES CONDITION (β = 0.03, *p* = 0.002, 95% CI = 0.01 to 0.05) and TASK (β = -0.03, *p* = 0.003, 95% CI = 0.01 to 0.04) showed significant main effects, but no interaction (Tables 2 and 3). With FES, step length increased by approximately 0.03 m during both ST and DT. During DT, step length decreased by 0.03 m without FES as well as with FES (Table 3).

Step width significantly decreased with FES (β = -0.91, *p* = 0.019, 95% CI = -1.66 to -0.15), but TASK did not show a significant effect (β = 0.39, *p* = 0.308, 95% CI = -1.14 to 0.37) (Tables 2 and 3). With FES, step width decreased by 0.91 cm during ST and by 1.12 cm during DT (Table 3). The non-significant comparison of ST vs. DT revealed an increase in step width by 0.60 cm without FES and 0.39 cm with FES.

### Kinematics

In terms of kinematics, small but significant effects were observed in maximum toe clearance and maximum knee flexion during swing phase.

Maximum toe clearance during swing increased with FES (β = 12.11, *p* = 0.002, 95% CI = 4.57 to 19.65), but no significant effect of TASK was found. In the ST condition, maximum toe clearance increased from 127.4 mm (± 36.4) without FES to 141.1 mm (± 39.5) with FES. In the DT condition, it increased from 123.7 mm (± 37.4) to 133.7 mm (± 39.8) (Tables 2 and 3).

For maximum knee flexion during swing, the TASK effect was small (β = -1.61, *p* = 0.024, 95% CI = 0.22 to 3.00), demonstrating a decrease in knee flexion during DT, regardless of without FES or with. Without FES, maximum knee flexion decreased from 55.4° (± 9.3) during ST to 54.3° (± 9.7) during DT. Similarly, with FES, maximum knee flexion decreased from 56.5° (± 9.8) during ST to 54.9° (± 10.6) during DT (Table 4).

All other analysed sagittal kinematic variables, including dorsiflexion at heel-strike, maximum dorsiflexion during swing phase, and minimum toe clearance at mid-swing, variables potentially influencing overall toe clearance and foot lift, showed no significant main effect or interaction effects (Tables 2 and 3).

### Cognition rate

The cognition rate was only measured during dual-task walking, allowing exclusively for the analysis of FES CONDITION effects. With FES, the cognition rate was significantly higher, with a mean difference of 0.25 (*p* = 0.005, 95% CI = 0.09 to 0.40), compared to without FES (Table 4).

### Excluded variables

Due to the lack of normally distributed data and clear statistical effects, further analysis was not performed for max. hip flexion during swing phase and coefficients of variation (CoV) for all represented variables. CoV was calculated by dividing the standard deviation by the mean and multiplying by 100. The only noteworthy result was a tendency toward higher step length CoV during dual-task walking.

## Discussion

Regarding the literature, FES has predominantly been applied as an isolated foot drop orthosis with the main focus on analysing immediate and therapeutic effects^6,9^, with only a few studies addressing complex gait tasks, such as stepping accuracy^38^ or obstacle avoidance^39,40^. However, we suggest that FES may contribute to broader effects of FES on gait function, stability and even the interplay between cognitive function and motor control effort.

In the present study, we investigated the effects of FES on single- and dual-task walking in patients with neurological conditions such as cerebral palsy, incomplete spinal cord injury, multiple sclerosis, traumatic brain injury, and hereditary spastic paraplegia with foot drop. Using a repeated-measures design, we analysed the main effects of FES CONDITION and TASK as well as the FES CONDITION*TASK interaction on participants’ gait and cognitive performance outcomes.

The results in Tables 2 and 3, and 4 show minor effects of FES on foot lift kinematics but greater effects on overall gait function, as reflected in spatio-temporal parameters, regardless of cognitive load. While dual-task walking led to worsened gait function, FES counteracted this decline and improved cognitive performance.

The TASK condition led to a worsening of most spatio-temporal parameters, except for step width, whereas FES improved these parameters, resulting in faster walking speed, longer step length, and narrower step width. Except for patients with cerebral palsy^14,17^ an increase in walking speed aligns with existing literature^9,19,20,23,41^ which goes together with higher step length. To our knowledge, there is currently no literature on effects of FES on step width. Our findings show that FES leads to a significant reduction in step width, suggesting a more stable and secure gait. One study on ankle-foot orthosis (AFO) demonstrated similar results in walking speed^42^, with faster walking under AFO use, irrespective of the TASK condition and without AFO*TASK interaction.

We found that FES generally enhanced toe clearance, though none of the analysed kinematic variables showed significant improvements. However, many studies report a significant increase in maximum dorsiflexion during swing^14,43^ and higher minimum toe clearance^14,41^. The lack of significant improvements in our study regarding dorsiflexion during swing could be attributed to factors such as cumulative effects across joints from both contralateral and ipsilateral sides, an underpowered sample size, or specific FES device limitations. Nevertheless, consistent with the tendencies observed in our pilot study^27^, we found no significant effect of FES on foot drop. The only significant kinematic variable identified was maximum toe clearance in swing, which is more indicative of a stable gait initiation for the next heel-strike rather than enhanced toe clearance during swing phase.

During dual-task walking with FES, subjects demonstrated a significant improvement in cognition rate compared to walking without FES. Indicating that subjects using FES could more effectively balance cognitive load while maintaining walking, which is crucial for environmental interactions. In existing FES literature on more complex gait tasks, only stepping accuracy and obstacle avoidance tasks have been studied^38–40^ and not specifically on additional cognitive load itself. Although both AFO and FES effectively improved walking speed, FES allowed patients after stroke to avoid obstacles significantly better than AFO^39,40^. Additionally, during a target accuracy task, medio-lateral accuracy improved significantly with FES^38^, aligning with our findings. With FES during dual-task walking, cognition rate improved significantly, alongside a decrease in step width.

Drake, et al.^42^utilised a similar study design with AFOs and dual-task walking in patients after stroke, although with a different cognitive task involving feedback on an auditory memory task. Participants using AFO increased their walking speed regardless of TASK condition, yet no significant cognitive improvement was observed. Even though, the cognitive task was different, the significant results of our study may suggest that FES offers superior cognitive-motor interference during dual-task walking compared to AFO. Future research is needed to examine cognitive-motor interference further especially in different dual-task conditions as well as with different treatment methods.

The underlying neurophysiological mechanisms by which FES enhances cognition rate and gait function remain unclear. The primary efferent task of FES is to supports or take over the foot lift execution and thereby indirectly reduce the motor control effort.

However, the altered afferent feedback may be of higher clinical relevance. By modifying proprioceptive input, FES can reduce cognitive and motor control effort by shifting motor control from motor cortex (M1) to sensory cortex (S1), making tasks like foot lifting less demanding. Enhanced sensory feedback allows M1 to rely more on S1 for movement execution which reduces the need for detailed motor planning^44^.

Furthermore, the afferent rhythmic feedback from FES may modulate the central pattern generator^45,46^ and thereby reduces the need for conscious control of walking and freeing up resources for additional (cognitive) tasks. Additionally, the afferent feedback may induce supraspinal neuroplasticity, resulting in increased voluntary motor control^10^, muscle volume and strength^24^ potentially reducing motor effort.

Most likely, these mechanisms interact, with afferent feedback prompting neuroplastic changes at spinal level for an automatic, unconscious gait and at supraspinal level for improved voluntary control and a reduced need for detailed motor planning, while the efferent stimulation ensures sufficient foot lift. Ultimately, even minor improvements from efferent stimulation in foot lift may be sufficient for adequate toe clearance and the freedom for the aforementioned beneficial afferent effects to evolve.

Overall, we account the accumulation of the effects in walking speed, step width and cognition rate as main clinical relevance for the subjects. For each parameter by itself, the effects of FES are relatively modest but for walking speed still in the clinically meaningful range of 0.05 to 0.082 m/s^47,48^ with an improvement in 0.07 m/s with FES during dual-task walking.

The primary focus of this study was to evaluate the impact of FES on gait and cognition. Unfortunately, due to feasibility constraints, a placebo-controlled sham condition could not be integrated in the clinical setting so potential placebo effects cannot be ruled out. Furthermore, since inclusion criteria focused on upper motor neuron damage, the cohort is rather heterogeneous regarding neurological disorders. The response to FES may therefore vary. However, the sample size is too small for stratification by aetiology.

While the stimulation settings are specifically adjusted to meet each subject’s needs to ensure individual benefits, this approach introduces variability with respect to pulse width, frequency, intensity, and timing of the FES. However, it was not the purpose of this work to analyse optimum settings for FES but to reflect best clinical practice ensuring effectiveness of FES and maximizing benefits for each individual.

For people with neurological disorders our findings suggest that FES not only improves spatio-temporal parameters and better controlled foot positioning but also enhances cognitive performance during dual-task walking. This is particularly relevant for individuals at increased risk of falling, who may benefit from a reduced need of attention to walking itself. The consistent effects of FES across both single- and dual-task walking highlight its robustness in improving gait function and stability, regardless of cognitive load. The improved cognition rate with FES during dual-task walking may reflect a reduced need to focus almost exclusively on walking, allowing for more interaction with the environment while maintaining steady gait kinematics. These results support FES as a promising rehabilitation tool to enhance gait function, stability, and cognitive performance in neurological patients with foot drop.

## Data Availability

The datasets generated and analysed during the current study are available from the corresponding author on reasonable request.
